# Machine Learning Diffuse Optical Tomography Using Extreme Gradient Boosting and Genetic Programming

**DOI:** 10.3390/bioengineering10030382

**Published:** 2023-03-21

**Authors:** Ami Hauptman, Ganesh M. Balasubramaniam, Shlomi Arnon

**Affiliations:** 1 Department of Computer Science, Sapir Academic College, Sderot 7915600, Israel; 2 Department of Electrical and Computer Engineering, Ben-Gurion University of the Negev, Be’er Sheva 8441405, Israel; shlomi@bgu.ac.il

**Keywords:** diffuse optical tomography, extreme gradient boosting, genetic programming, inhomogeneous breast, inverse problems

## Abstract

Diffuse optical tomography (DOT) is a non-invasive method for detecting breast cancer; however, it struggles to produce high-quality images due to the complexity of scattered light and the limitations of traditional image reconstruction algorithms. These algorithms can be affected by boundary conditions and have a low imaging accuracy, a shallow imaging depth, a long computation time, and a high signal-to-noise ratio. However, machine learning can potentially improve the performance of DOT by being better equipped to solve inverse problems, perform regression, classify medical images, and reconstruct biomedical images. In this study, we utilized a machine learning model called “XGBoost” to detect tumors in inhomogeneous breasts and applied a post-processing technique based on genetic programming to improve accuracy. The proposed algorithm was tested using simulated DOT measurements from complex inhomogeneous breasts and evaluated using the cosine similarity metrics and root mean square error loss. The results showed that the use of XGBoost and genetic programming in DOT could lead to more accurate and non-invasive detection of tumors in inhomogeneous breasts compared to traditional methods, with the reconstructed breasts having an average cosine similarity of more than 0.97 ± 0.07 and average root mean square error of around 0.1270 ± 0.0031 compared to the ground truth.

## 1. Introduction

Imaging with randomly scattered light is a significant challenge with a pressing need in non-invasive biomedical diagnosis [1,2,3,4,5,6]. One of the more significant applications is a non-invasive functional imaging technique called diffuse optical tomography (DOT), which uses near-infrared (NIR) light to map in 3D the optical characteristics of tissue by penetrating it deeply [7,8,9,10,11,12,13]. Soft tissue, including the breast and the brain, can be penetrated several centimeters by diffuse light in the NIR wavelength range. Measurements from reflected or transmitted light at the tissue surface are used to reconstruct DOT images using inverse problems [14,15,16]. Moreover, DOT has many advantages, including the non-ionizing nature of the light spectra used for imaging tissues and the non-invasive nature of the technology. As a result, DOT has found critical applications, especially in breast tissue imaging [8,15,17] and tissue property estimation [10,18,19,20,21]. However, many challenges persist. Although near-infrared (NIR) photons can travel several centimeters into the tissue to enable non-invasive biomedical imaging, these photons scatter widely and migrate in random directions before escaping or being absorbed by the medium, making imaging and detection tasks challenging [20,22].

Moreover, DOT reconstruction is an ill-defined and inadequately framed topic that calls for an inversion regularization to enhance convergence [15,23]. Inaccurate DOT reconstruction can also be caused by model inadequacies, including poor boundary conditions, the chest wall beneath the breast tissue, and inaccurate background tissue estimation [8,24]. Due to the above reasons, DOT for breast cancer imaging has previously been investigated but found to be facing various challenges when considering large-scale medical deployment. Since then, significant technological advancements have been made, including improved computational efficiency that stimulated the creation of novel deep learning algorithms and a deeper comprehension of how light travels through tissues. Therefore, a less complicated yet computationally effective approach is needed to detect anomalies and tumors present in thick tissues.

Recent research has attempted to address some of the challenges posed due to the diffuse nature of photons and ill-posed inverse problems by employing deep learning (DL) [15,17,20,25,26] and shallow machine learning (ML) techniques [8,27]. The usage of ML algorithms regularly beats the analytical solution technique in all of the articles previously mentioned. Compared to traditional analytical models, ML and DL algorithms are better posed, faster, and provide a better convergence while determining tissues’ optical properties or detecting locations of tumors and anomalies in thick tissues. For instance, Murad et al. [28] experimentally demonstrated the simultaneous reconstruction of the absorption and scattering coefficient of tissue mimicking using a 1D convolution neural network (1D-CNN). Here, simple batch normalization (BN) layers were used to significantly improve the accuracy and reduce the time consumed for DOT image reconstruction. Yoo et al. [15] developed a novel DL algorithm that accurately detects anomalies in beast tissues by inverting the Lippman–Schwinger equation, achieving significant results. In a more recent study, Mozumder et al. [29] employed a model-based DL approach to improve the estimation of the absorption and scattering coefficient of diffuse media. It was shown in this study that the proposed DL method also significantly reduces computation time. Moreover, several excellent reviews [30] and tutorials [14] exist that provide valuable insights regarding the use of DL for DOT image reconstruction.

However, despite the improvements in accuracy obtained in DOT reconstruction by DL methods, there is still a pressing need for algorithms that provide similarly good results with smaller datasets and reduce the computational load on the system. ML algorithms may provide solutions to some of these issues as they work well on smaller datasets, can function without a GPU, and are generally computationally quicker than DL methods. Recently, Zou et al. [8] used a machine learning model with physical constraints to reconstruct DOT images. Yun Zou et al. proposed that the ML algorithm significantly reduced the DOT image reconstruction error, especially in high-contrast samples, compared to the Born gradient descent analytic reconstruction method. The significant observations and the results obtained from this work clearly show the potential of machine learning algorithms where DOT reconstruction problems are concerned.

Extreme gradient boosting or XGBoost is a widely used machine learning algorithm known for its high performance and accuracy in various applications such as regression, classification, and ranking problems [31]. XGBoost shows high scalability, robustness, and speed compared to other models [32,33]. Therefore, in this article, for the first time, to the best of our knowledge, we use XGBoost to detect tumors located in compressed breast tissues. We later apply an optimization method called genetic programming (GP) [34,35,36] to improve our results further. GP is an evolutionary algorithm that uses natural selection to evolve solutions to optimization problems. It can automate the design and optimization of complex systems, generate novel solutions, find robust and interpretable solutions, and be more efficient than other optimization algorithms. The proposed machine learning diffuse optical tomography (ML-DOT) method requires short training times and delivers high accuracy in detecting tumors even with small datasets, and thus significantly reduces the computational load.

The article is designed as follows: The imaging geometry used in this study and the data generation are described in Section 2.1, and the proposed technique for tumor detection in compressed breasts is described in Section 2.2 and Section 2.3, along with the obtained results. Section 3 discusses the results of the proposed algorithm, and we finish with the conclusions derived from this study in Section 4.

## 2. Materials and Methods

Image reconstruction in DOT has traditionally been achieved using inverse problems, which take the form [37]:

(1) Findx∈X from data y=A(x)+δ, y∈Y

where the parameter space is defined by X, Y is the measurement space, A is the propagation model of photons convolved with the optical component response, and δ is the noise mechanism in the system. The scarcity of ballistic photons coupled with the loss of imaging data as a result of repeated scattering events, however, results in a non-linear, ill-posed inverse problem, needing an effective solution to accomplish this challenging task. In order to simplify the DOT system and precisely locate cancers embedded in the compressed breast tissue, approaches based on extreme gradient boosting and genetic programming are proposed in this section. This section includes a brief introduction to the proposed machine learning techniques, the proposed methodology, and the results obtained from this study.

### 2.1. Simulating Photon Migration in Digital Breast Phantoms to Generate Dataset

The compressed breast geometry is chosen for this study. This particular type of geometry is used as it considerably reduces movement artifacts, evenly spreads the different layers of tissue, and also helps considerably reduce the amount of tissue to be imaged [16,38]. The dataset used to train, validate, and test the algorithm is created using the finite element method (FEM) [39]. The evaluation of DOT models is usually conducted through computer simulations, as it allows for easy comparison to the work of other scientists, creating a larger dataset and providing a better-controlled environment. Additionally, simulations allow for the avoidance of costly and wasteful fabrication of clinical prototype systems that may possess inherent engineering problems. Furthermore, it is both complicated and expensive to create an inhomogeneous breast phantom for experimental purposes [14,38]. The compressed breast mesh itself is loaded from the “DigiBreast” [40] digital breast phantom modeled accurately using the optical properties of the skin, breast tissues, and the chest wall [17,41]. The digital breast phantom includes a realistic 3D glandularity map, which is more detailed than the representation of breast tissue in conventional numerical breast phantoms. These traditional phantoms use piecewise-constant regions to represent the fibroglandular and adipose tissue, which can result in a loss of information. In contrast, the digital breast phantom uses statistical or fuzzy segmentation methods to create spatially varying tissue volume fraction maps, which preserve more detailed information about the breast tissue.

**Figure 1:**
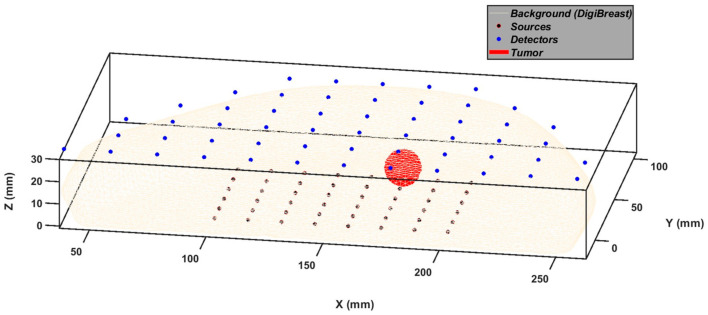
Simulated compressed breast mesh with various components and source–detector geometry.

The dimensions of the compressed breast are 220.8 mm, 102.9 mm, and 23.7 mm in the *X*, *Y*, and *Z* directions, respectively. A parallel-plate-based measurement scheme is employed to set a 48 × 54 source–detector arrangement on either side of the breast. The simulated compressed breast mesh and the source–detector arrangement are shown in Figure 1.

The simulations are then performed in the transmission geometry using the Toast++ [39] forward solver to simulate light intensity measurements at the detectors. As tumors often have a high blood concentration, in this study, they are modeled as oxyhemoglobin spheres with radii ranging from 2 mm to 15 mm randomly located inside a compressed breast mesh. The algorithm used 5000 simulated breast measurements taken in the transmission geometry as input, and the *x*, *y*, and *z* coordinates, the absorption coefficient matrix, and the tumor’s radius were the labels. These simulated measurements were performed in the continuous wave regime at a wavelength of 833 nm, and a 2% gaussian noise was added to mimic errors that might occur if the measurements were taken from an experimental procedure.

### 2.2. Detecting Tumors in the Compressed Breast Using Extreme Gradient Boosting

Following the creation of the dataset, the collected data are used to solve the inverse problem and detect the location of tumors inside the compressed breast. A machine learning algorithm called extreme gradient boosting (XGBoost) is employed to solve the inverse problem. XGBoost is a distributed, scalable gradient-boosted decision tree (GBDT) machine learning framework with several applications that use regression [32] and classification [33]. Here, gradient boosting refers to “boosting” or strengthening one weak model by fusing it with several other weak models to create a more robust model. As an extension of boosting, gradient boosting formalizes the process of additively creating weak models as a gradient descent method over a fitness function. Gradient boosting algorithms create a model that predicts the label by evaluating a tree of logical feature questions and determining the minimum number of questions required to assess the probability of obtaining a correct decision. To reduce errors, gradient boosting establishes desired outcomes for the upcoming model. The gradient of the error concerning the prediction determines the targeted outcomes for each case, hence the name “gradient boosting”.

Created by Tianqi Chen and contributions from numerous developers, “Extreme Gradient Boosting (XGBoost)” is one of the most important and successful implementations of gradient boosting machines [31]. XGBoost is a scalable and highly accurate gradient-boosting algorithm that pushes the limits of computing power for boosted tree algorithms. It was created primarily to enhance machine learning models’ performance and computational speed. Unlike GBDTs, where decision trees are generated sequentially, the trees in XGBoost are built in parallel. The XGBoost method takes a level-wise approach, scanning over multiple gradient values and using the partial sum generated from the gradient values to evaluate the performance of the splits for each possible split in the training dataset.

**Figure 2:**
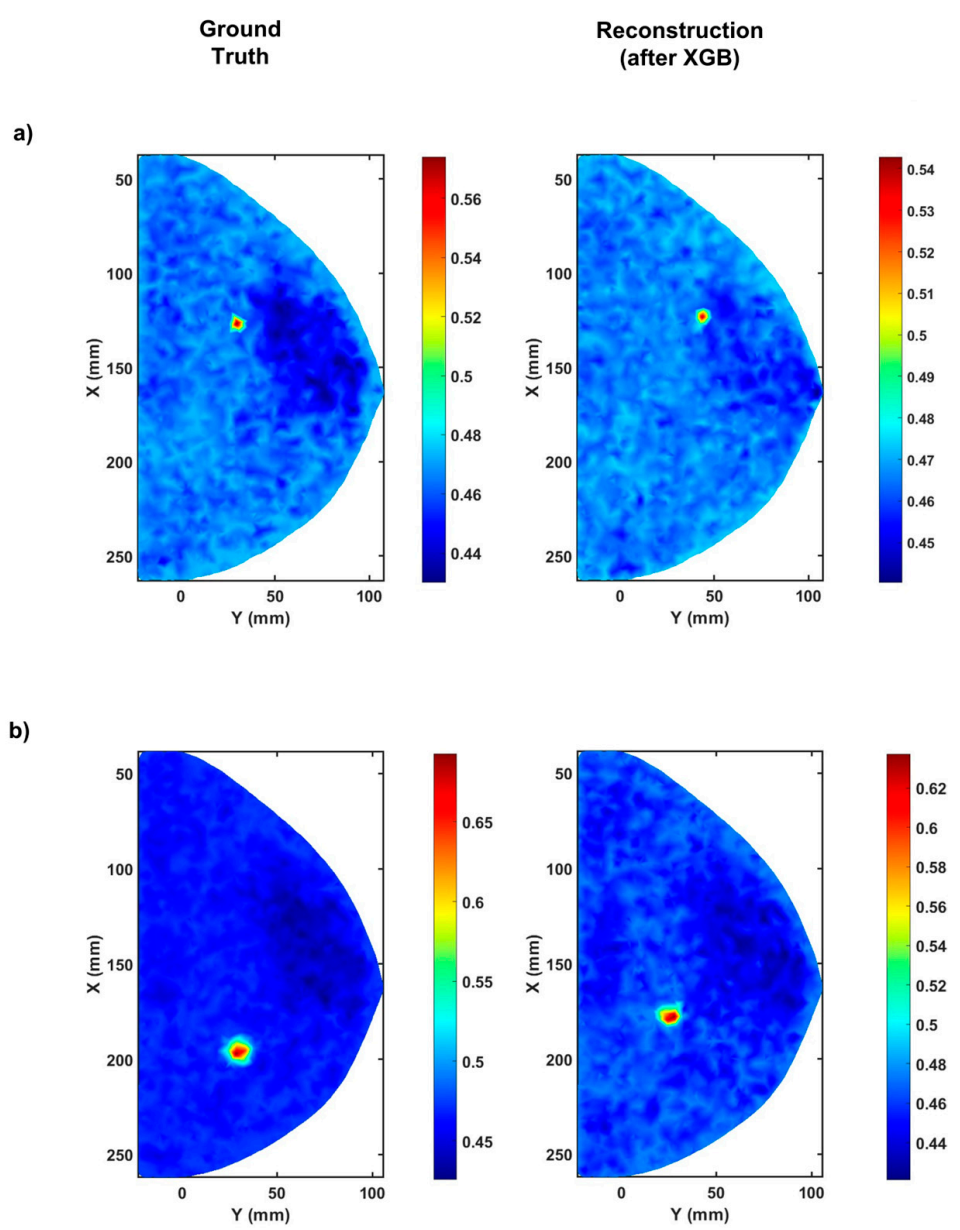
The reconstructed tumor locations using predictions from the XGBoost machine learning algorithm. (**a**) showing the reconstructed tumor locations at (*z* = 5.325 mm), and (**b**) showing the reconstructed tumor locations at (*z* = 11.289 mm), are two different examples of the XGBoost predictions. The color bar shows the absorption coefficient in (${\mathrm{cm}}^{-1}$).

As described earlier, the dataset is created using 5000 different measurements at the transmission boundaries of the compressed breast. The log of the measured signal at each detector with respect to different sources (log(d)) is used as the input to the XGBoost algorithm. The “*x*”, “*y*”, and “*z*” coordinates, the absorption coefficient, and the radius are the labels. The algorithm is employed to find an accurate model that establishes a precise relation between the simulated measurements and the tumor locations. The root mean squared error (RMSE) loss function is used to assess the algorithm’s performance, and the accuracy of the predicted outcome is measured using the RMSE and the cosine similarity (CS) metrics [42]. The RMSE loss function is given by:

(2) $$\mathrm{RMSE}=\sqrt{\overset{N}{\underset{i=1}{\sum }}\frac{{\left(Y_{i}-X_{i}\right)}^{2}}{N}}\;$$

and the cosine similarity is given by:

(3) $$\mathrm{CS}=\frac{\sum_{i=1}^{N}X_{i}Y_{i}}{\sqrt{\sum_{i=1}^{N}X_{i}^{2}}\sqrt{\sum_{i=1}^{N}Y_{i}^{2}}}\;$$

where $Y_{i}$ and $X_{i}$ are the predictions and the labels, respectively, and *N* is the total number of datasets. These metrics are specifically used primarily because the R^2^ metrics perform relatively poorly when the prediction window is narrow, as is the case with the radius of the spheres. Furthermore, the RMSE and the CS metrics are better suited to solving inverse problems where the prediction interval is narrow and continuous [8,43]. A computer with an i9 series 9900k processor and two NVIDIA GeForce RTX 2080Ti graphics processors were used to build and train the XGBoost algorithm. Each GPU contains 11 GB of VRAM, and the two GPUs are connected through an NVlink. The network is by Adam Optimizer with an initial learning rate of 0.0001. For the XGB predictions, 50% of the total dataset is used, out of which 60% is used for training and validation, and 40% of the data is used for testing. The XGBoost algorithm learns the features from the input and the labels and accurately predicts the relationship between the input (measurement data) and the labels (tumor location) by regression. Due to variations in the dimensions of the compressed breast and the nature of the coordinate system used to model the compressed breast, the model prediction is made separately for the three different coordinates, the absorption coefficient, and the radius. The best results obtained by the XGBoost algorithm are RMSE values of 0.1862 ± 0.0018 for the *x* coordinate, 0.1678 ± 0.0042 for the *y* coordinate, 0.1505 ± 0.0009 for the *z* coordinate, 0.1131 ± 0.0091 for the absorption coefficient, and 0.2157 ± 0.0103 for the radius. Some of the predictions of the XGBoost algorithm are shown below in Figure 2.

From the results obtained by the XGBoost algorithm, it is undoubtedly well-posed to detect tumors to a certain degree of accuracy using the simulated measurements. The predictions by the XGBoost algorithm obtained a very high average cosine similarity value of 0.9564 ± 0.0076 compared to the ground truth. However, the predicted size and locations of the embedded tumors could still be improved, especially the predictions of the radius and the *z* coordinate, which is also the mean direction of propagation. Therefore, the following section applies a post-processing algorithm using genetic programming (GP) to improve the model predictions.

### 2.3. Enhancing Tumor Detection Capabilities Using Genetic Programming

**Figure 3:**
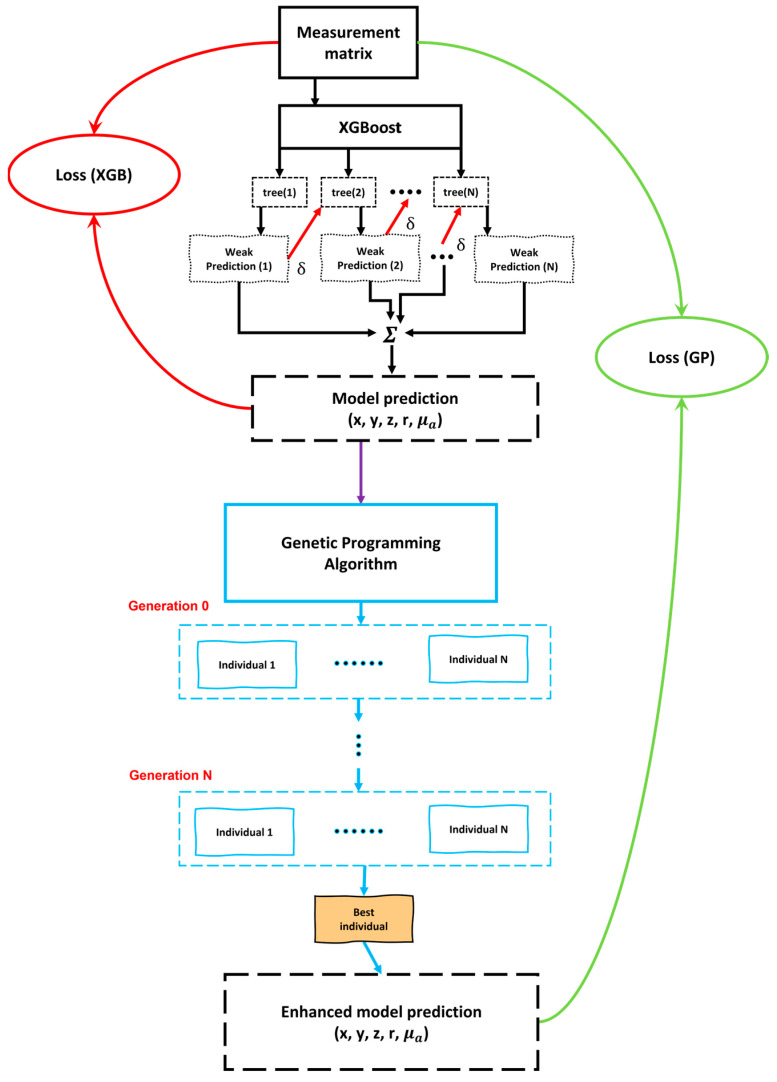
Schematic block diagram showing the workflow of the proposed methodology. The black and blue boxes show the working of the XGBoost and GP algorithms, respectively.

Genetic programming (GP) is a nature-inspired hyper-heuristic search algorithm that optimizes a set (or a population) of solutions (or individuals), embodied as computer programs, which are typically represented as tree-like structures, using a predefined loss function (or a fitness function) for a given task [35,44]. In other words, GP starts with a high-level declaration of “what needs to be done” and constructs a computer program to solve the problem automatically. As described in Figure 3, GP begins with a primordial soup of a large pool of randomly generated computer programs (initial guess of the model prediction), and this population of programs evolves throughout generations. The evolutionary search employs the Darwinian concept of natural selection (survival of the fittest) and analogs of many naturally occurring events, such as crossover (sexual recombination), mutation, gene duplication, and gene deletion. Moreover, genetic algorithms, by means of fitness-based selections and applying genetic operators over time, can optimize solutions automatically during the course of simulated evolution. Thus, GPs’ adaptive nature, free of human prejudices or biases, may often provide answers better than the most exemplary human efforts.

Genetic or evolutionary algorithms have a wide range of applications, particularly in domains where an exact structure of the solution is unknown in advance or when finding an approximate solution is considered suitable [45]. Genetic programming is frequently used in conjunction with other types of machine learning because it is useful for performing symbolic regressions and feature classifications [46].

The execution of a GP algorithm is described below:

1. Randomly generate an initial population of solutions called individuals. Each individual is generated as a random tree of limited depth, consisting of nodes taken from the terminal set and the function set. The terminal set contains constants and variables, and the function set consists of various operators, for example, mathematical operations, logical operators, etc.
2. While the termination criterion is not fulfilled, the following sub-steps are repeated:
  a. Evaluate the individuals in the current population according to the fitness function, which outputs a numerical value representing the quality of the individual as a solution.
  b. Select individuals from the population using a selection method, where the probability for selection is related to fitness values, for producing the next set of individuals.
  c. Apply the following genetic operators to produce new individuals with predetermined probabilities:
    I. Reproduction: clone an individual selected by the sub-step ”b” to the population.
    II. Crossover: randomly recombine two selected individuals to produce two new offspring.
    III. Mutation: randomly alter one selected individual to produce one new offspring.
3. Output the best individual found during the run as the output.

To implement the steps described above, we employed Koza-style [47] genetic programming using the DEAP Python environment [48]. We now describe the details of our implementation:

Essentially, each GP individual represents an ℝ→ℝ function, which receives a single real-valued coordinate as input (namely, x, y, z, or radius) outputted by XGBoost in the previous stage), and outputs a single real-value, as an estimate for the actual coordinate (x, y, *z*, or radus). The function set for each individual (as can be seen in the pseudo-code above) is comprised of binary arithmetic operations. The terminal set is comprised of a single real-valued coordinate and integer ephemeral random constants (ERCs) [35,44,47] in the range [−5, 5]. For example, if the coordinate in question is x, it is possible to form an expression such as add (multiply (*x*, *x*), negate (multiply (*x*, 5.0))) representing the function: $x^{2}+\left(-5x\right).$ Typically, GP trees can represent expressions that are far more complex.

For assessing our individuals, fitness was calculated by randomly selecting 10% of the dataset (with the given single label) and applying the individual to each of the selected examples. The fitness score was determined by the RMSE measure between the actual values for the labels in the dataset and those predicted by the individual. We used tournament selection as the selection mechanism. Additional parameters of the GP algorithm are given in Table 1, and the entire workflow of the proposed method, including the XGBoost and GP, is shown in Figure 3.

**Figure 4:**
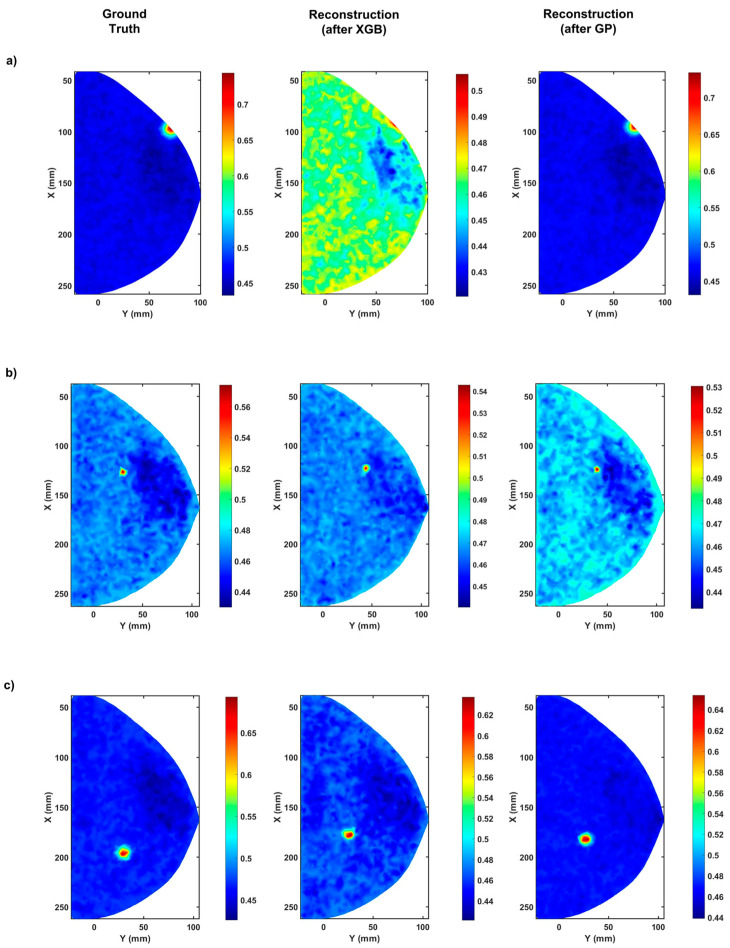
The reconstructed tumor locations using predictions from the GP post-processing algorithm. (**a**) showing the reconstructed tumor locations at (*z* = 14.375 mm), (**b**) showing the reconstructed tumor locations at (*z* = 5.325 mm), and (**c**) showing the reconstructed tumor locations at (*z* = 11.289), are different examples of the XGBoost and GP predictions. The color bar shows the absorption coefficient in (${\mathrm{cm}}^{-1}$).

The remaining 50% of the total data after the XGBoost is used for the GP algorithm. The data split for the GP algorithm’s training, validation, and testing is similar to the data split used for the XGBoost, and the algorithm is also run on the same computational hardware. After applying the algorithm for 30 runs with 100 generations each, the final results obtained after XGBoost and GP are RMSE values of 0.1808 ± 0.0014 for the *x* coordinate, 0.1539 ± 0.0057 for the *y* coordinate, 0.1340 ± 0.0032 for the *z* coordinate, 0.0975 ± 0.0065 for the absorption coefficient, and 0.2017 ± 0.0126 for the radius. The reconstructed images from the predictions are shown in Figure 4.

As seen in Figure 4, predictions significantly improve when the post-processing GP algorithm is employed. The most significant enhancement is observed in the predictions of the *z* coordinate of the embedded tumors. Considering that the z-axis is the mean direction of the photon propagation due to the forward scattering anisotropy of the media, this is a commendable result, as minimal errors in the prediction of the *z* coordinate will allow us to detect anomalies and tumors at greater depths. The results obtained using the XGBoost and the enhancement due to GP are summarized in Table 2.

## 3. Discussion

As seen in Figure 4, the predicted outcomes are very close to the ground truth locations and the radii. When the CS metric is used to validate the model’s performance, an average similarity of 0.9720 ± 0.0062 is observed when the predicted labels are compared to the ground truth. From the RMSE and CS values, it is apparent that the prediction is very robust. The algorithm is also computationally fast, as training consumes less than 60 s. It must also be noted that the algorithms do not have any prior information or knowledge regarding the inverse solution to DOT. Our study found that our predictions for the *x* and *y* coordinates were significantly more accurate than those for the *z* coordinate and radius. This discrepancy may be due to the fact that the range of the *z* coordinate and radius was limited compared to the range of the *x* and *y* coordinates. This limited range not only adversely affected the algorithm’s performance but also negatively impacted the simulated measurements. The small changes within this limited range were not as clearly reflected in the measurement matrix, resulting in a less accurate regression model for predicting the tumor location and radius.

Moreover, our study’s absorption coefficient reconstruction is still not very accurate. The XGBoost algorithm underestimates the absorption coefficient, while the GP regularly overestimates the absorption coefficient. This is likely due to the inhomogeneous distribution of tissue within the breast and the varying contrasts between the anomalies (such as tumors) and the surrounding tissue. Inhomogeneity in the tissue distribution can introduce variations in the optical properties of the breast, leading to challenges in accurately reconstructing the absorption coefficient. Similarly, the variability in contrast between the anomalies and the background can make it difficult to accurately distinguish between the two, leading to errors in the reconstruction. These factors likely contributed to the limitations in the accuracy of the absorption coefficient reconstruction in our study.

Despite these limitations, it is worth noting that the algorithm was still able to learn an accurate model for solving the inverse problem and identifying tumors based on a relatively small, simulated dataset. This demonstrates the robustness and effectiveness of the algorithm, which effectively processed and analyzed the data despite the challenges presented by the limited range of the *z* coordinate and radius. Overall, our results show that the combination of XGBoost and genetic programming (GP) can be a powerful tool for solving inverse problems in diffuse optical tomography and may have potential applications in medical imaging and other fields.

XG boost and genetic programming (GP) are highly effective algorithms for solving diffuse optical tomography (DOT) problems. XG boost, a decision tree-based algorithm, is particularly well-suited for handling highly non-linear data and can be used for classification and regression tasks. GP is versatile and can be used for a variety of purposes, including regression, classification, and optimization. These algorithms have been shown to outperform other approaches, such as conventional analytical inverse problems and convolutional neural networks (CNNs), in many complex applications due to their ability to accurately process and analyze the data. Therefore, XGBoost and GP are the preferred choice for solving DOT problems.

Moreover, the primary issue with employing neural networks to solve DOT inverse problems is the near impossibility of collecting massive amounts of experimental data to train the algorithm, leading to trade-offs between precision and accuracy in actual investigations. This study addresses this issue as shallow machine learning algorithms such as XGBoost do not require substantial amounts of data for accurate model prediction. The comparison of the effectiveness of our algorithm with that of the existing state-of-the-art algorithms is shown in Table 3. A more detailed comparison to other imaging modalities and algorithms using different metrics can be found in excellent review articles [14,30,49].

As seen in Table 3, the proposed method is well-suited and outperforms other algorithms that solve a similar problem. Furthermore, transfer learning can close the gap between simulations and real tissue imaging. Additionally, to improve performance, the proposed methods can be trained using a more complex and realistic dataset with different geometries and source–detector configurations, paving the way for the realization of large-scale medical applications involving non-invasive and radiation-free diffuse optical techniques that can provide an accurate diagnosis.

## 4. Conclusions

In this study, an extreme gradient boosting algorithm was used in conjunction with a genetic programming algorithm to detect embedded tumors or anomalies in inhomogeneous compressed breast tissues. A dataset of 5000 compressed breasts with anomalies was simulated for the study, and simulated light measurements were used to determine the location and size of the tumors. After applying the proposed method, the obtained RMSE and cosine similarity values showed that the algorithm was highly accurate and robust when the predicted tumor locations and sizes were compared to the ground truth. Moreover, the issues regarding large datasets and expensive computational costs were addressed to a certain extent as only a few breast samples were used, and the tumor location and radius predictions consume a very minimal amount of time. The results from the proposed method to accurately determine the anomalies in breast tissues show that extreme gradient boosting and genetic programming algorithms can be employed to detect tumors in compressed breast tissues with excellent accuracy compared to previously existing methods.

## Figures and Tables

**Table 1:** Parameters for the GP algorithm.

Parameter	Range of Values
Population size	Between 10,000 and 15,000
Generation count	Between 100 and 250
Reproduction probability	0.35
Crossover probability	0.5
Mutation probability	0.15 (including ERC)
Tree depth	Between 2 and 6
Tournament size	4

**Table 2:** Performance of the proposed model in terms of RMSE.

Values (Units)	RMSE (After XGBoost)	RMSE (After GP)
X coordinate (mm)	0.1862 ± 0.0018	0.1808 ± 0.0014
Y coordinate (mm)	0.1678 ± 0.0042	0.1539 ± 0.0057
Z coordinate (mm)	0.1505 ± 0.0009	0.1340 ± 0.0032
Radius (mm)	0.2157 ± 0.0103	0.2017 ± 0.0126
$\langle \mu_{a}\rangle \;$(mm^−1^)	0.1131 ± 0.0091	0.0975 ± 0.0065

**Table 3:** Comparison of the XGBoost-GP algorithm with existing algorithms in terms of RMSE.

S. No.	Article	Research Type	Background Type	RMSE
P.	**Proposed algorithm (XGBoost + GP)**	Simulation	**Inhomogeneous background mesh (DigiBreast [40])**	**0.12**
1.	Jaejun Yoo et al. [15] (Neural network for inverting Lippman–Schwinger equation)	Simulation and Experiment	Homogeneous background mesh (breast mesh and full body rat mesh)	0.66
2.	Yun Zou et al. [8](ML-PC model)	Simulation and Experiment	Homogeneous background mesh	0.30
3.	GM. Balasubramaniam et al. [17] (Cascaded feed-forward neural network)	Simulation	Homogeneous background mesh	0.17

## Data Availability

The simulation and machine learning codes presented in this paper are not publicly available but may be obtained from the authors upon reasonable request.
